# Efficient Heterologous Production of *Rhizopus oryzae* Lipase via Optimization of Multiple Expression-Related Helper Proteins

**DOI:** 10.3390/ijms19113372

**Published:** 2018-10-28

**Authors:** Liangcheng Jiao, Qinghua Zhou, Zhixin Su, Yunjun Yan

**Affiliations:** Key Laboratory of Molecular Biophysics, the Ministry of Education, College of Life Science and Technology, Huazhong University of Science and Technology, Wuhan 430074, China; jiaoliangcheng@gmail.com (L.J.); qinghuazhou@hust.edu.cn (Q.Z.); xuzhixin5512@163.com (Z.S.)

**Keywords:** *Rhizopus oryzae* lipase, heterologous overexpression, *Pichia pastoris*, helper proteins, high-density fermentation

## Abstract

This study is dedicated to efficiently produce *Rhizopus oryzae* lipase (ROL) by optimizing the expression of multiple expression-related helper proteins in *Pichia pastoris*. A series of engineered strains harboring different copy numbers of the *ROL* gene and different copies of the chaperone *Pdi* gene were first constructed to examine the influence of *Pdi* gene copy number on ROL production. The results showed that multiple copies of *Pdi* gene did not significantly improve ROL expression. Then, the effect of the co-overexpression of 10 expression-related helper proteins on ROL secretion was investigated by screening 20 colonies of each transformants. The data from shaking-flask fermentation suggested that Ssa4, Bmh2, Sso2, Pdi, Bip, Hac1, and VHb had positive effects on ROL expression. Subsequently, Ssa4, Bmh2, and Sso2, which all participate in vesicular trafficking and strongly promote ROL expression, were combined to further improve ROL production level. ROL activity of the screened strain GS115/5ROL-Ssa4-Sso2-Bmh2 4# attained 5230 U/mL. Furthermore, when the helper proteins Pdi, Bip, Hac1, and VHb were individually co-expressed with ROL in the strain GS115/5ROL-Ssa4-Sso2-Bmh2 4#, lipase activity increased to 5650 U/mL in the strain GS115/5ROL-Ssa4-Sso2-Bmh2-VHb 9#. Additionally, the maximum ROL activity of 41,700 U/mL was achieved in a 3 L bioreactor for high-density fermentation via a sorbitol–methanol co-feeding strategy, reaching almost twofold the value of the initial strain GS115/pAOα-5ROL 11#. Thus, the strategies in this study significantly increased ROL expression level, which is of great potential for the large-scale production of ROL in *P. pastoris*.

## 1. Introduction

Lipases (triacylglycerol acylhydrolases, EC 3.1.1.3) can catalyze hydrolysis, ester synthesis, and trans-esterification processes at the oil–water interface [1,2]. These special functions of lipases have endowed them with a great biotechnological potential. They are now widely used in a variety of industrial areas, such as for flavor, detergents, pharmaceutical, bioremediation, and biodiesel preparation [3,4]. As a noticeable enzyme, *Rhizopus oryzae* lipase (ROL) possesses strong 1-, 3-regiospecificity, which has been extensively used to produce biodiesel [5,6]. However, as known, the yield of ROL in the original *R. oryzae* strain is too low to meet the huge demands of the industry. Recently, ROL has achieved relatively high secretion levels via efficient heterologous expression in *Pichia pastoris* [7,8], but its expression level still needs to be further improved so as to greatly reduce the production cost. Thus, it is urgent to develop more effective strategies.

The *P. pastoris* expression system is widely applied for high-level expression of various heterologous proteins owing to its ease of genetic operation, its ability to grow to a high cell concentration under low nutritional conditions, its capability to execute complex eukaryotic post-translational modifications, its multiple promoters available, especially its alcohol oxidase 1 promoter (pAOX1), which is tightly regulated by methanol [9,10,11]. As known, the correct secretion of proteins must undergo the processes of transcription, translation, folding, and secretion. A series of factors can affect the expression level of proteins in *P. pastoris*, including promoter strength, gene dosage, secretion signal, protein translation, protein assembly, protein secretion, and fermentation conditions [12,13,14]. Among them, one of the most effective strategies to enhance protein production is to screen strains containing multiple copies of the target gene [14,15]. Nevertheless, as the gene dosage increases, recombinant proteins may excessively accumulate in the endoplasmic reticulum (ER), causing cellular stress and then activating the unfolded protein response (UPR), which would trigger the splicing of *Hac1* mRNA and generate the transcriptional activator Hac1 [16,17]. Next, the UPR activator Hac1 would reduce cellular stress by regulating several downstream genes involving in protein folding and transport, ER quality control, and ER-associated degradation (ERAD) [16,18]. However, the cells′ intrinsic regulatory mechanisms do not always remove cell stress effectively. To further increase protein secretion levels, some reported strategies trying to aid proteins folding as well as to reduce ER stress, have successfully achieved a higher level of gene expression. Among these, are the co-expression of the chaperones immunoglobulin-binding protein (Bip), protein disulfide isomerase (Pdi), and/or calnexin-like protein 1 (Cne1) [19,20] and the increased expression of the UPR activator Hac1 [7] and of ER oxidoreduction 1 (Ero1) [21]. In addition, the phosphomannomutase Sec53, which participates in both protein folding and ER quality control, has also been successfully employed to enhance protein production [20].

Protein secretion must go through the protein transport process, which also affects protein secretion. Several transport-related proteins, such as the cytosolic chaperone Ssa4, which is responsible for the transport of target nascent proteins to the ER membrane [22], and 14-3-3 protein Bmh2, which is involved in protein exit from the ER [23], have been reported to increase protein expression levels in *P. pastoris* [20,22]. Another secretion helper factor, Sso2, acting as one of the soluble *N*-ethylmaleimide-sensitive factor attachment protein receptors and helping the fusion of secretory vesicles at the plasma membrane [24], could also achieve the purpose of enhancing protein production [22].

When growing under high cell density conditions, *P. pastoris* requires a large amount of oxygen to maintain cell growth. In addition, oxygen solubility would be gradually reduced as the cell density increases [25]. So, oxygen intake becomes a bottleneck in protein expression under high-density conditions. To alleviate this problem, vitreoscilla hemoglobin (VHb) from *Vitreoscilla stercoraria*, which can facilitate oxygen delivery, has been used to promote protein expression [26].

Although the above-mentioned strategies have successfully increased the expression levels of some proteins, they cannot be effective for all proteins. For example, the secretion of glucose oxidase (GOD) was remarkably enhanced via co-expression of Sec53 [20]. However, the overexpression of Sec53 did not increase the production of Δ^9^-tetrahydrocannabinolic acid synthase [27]. Thus, the effectiveness of these strategies for the expression of a specific protein, including ROL, may be uncertain. So far, some strategies have been successfully applied to enhance ROL production in *P. pastoris*. For example, the ROL production level was effectively improved by co-overexpression of Ero1 and Pdi [8]. The co-expression of *Saccharomyces cerevisiae* Hac1 resulted in a threefold enhancement in ROL expression levels [7]. Moreover, ROL activity was improved 15.8-fold via a strategy combining the optimization of gene copy number with the co-expression of ERAD-related proteins [14]. However, there is no systematical optimization of the above-mentioned expression-related helper proteins to improve ROL expression levels. This optimization could help us to find a more appropriate expression strategy leading to a significant increase in ROL production.

Therefore, in this study, based on a previously constructed gene copy number-optimized recombinant strain GS115/pAOα-5ROL 11# [14], we first investigated the effect of 10 helper factors on ROL production in *P. pastoris*. Then, the helper genes which showed a positive effect on ROL production were selected and combined together to further enhance ROL secretion levels. Moreover, a 3 L high-density fermentation of the optimized strains was performed to test the maximum capability of ROL secretion.

## 2. Results

### 2.1. Influence of the Co-Expression of Different Copies of Pdi on ROL Production

Previously, we created a series of strains harboring different gene copy numbers of *ROL* and found that *ROL* gene dosage had an important effect on ROL expression [14]. To investigate the effect of the gene copy number of the expression-related helper factors on ROL production, Pdi, being an important ER chaperone, was selected as the model to achieve this goal.

**Figure 1:**
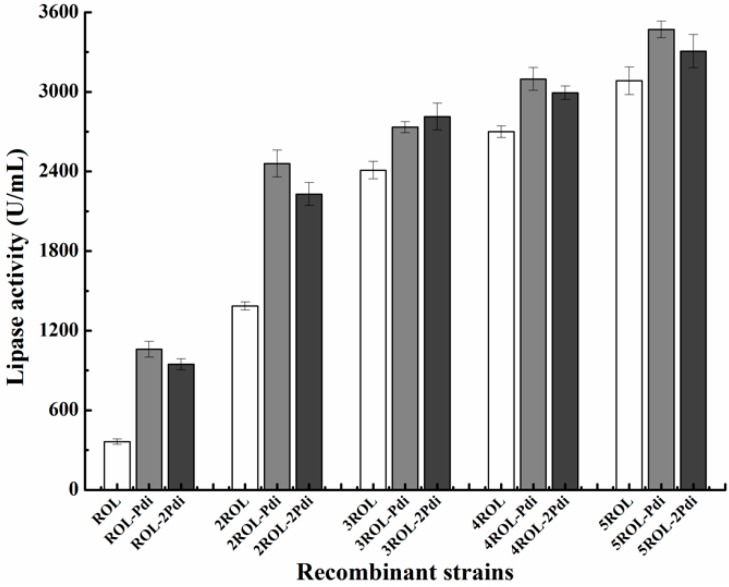
*Rhizopus oryzae* lipase (ROL) activity of the recombinant strains harboring different copy numbers of *ROL* gene and protein disulfide isomerase (*Pdi*) gene. All values are presented as the mean ± standard deviation of three independent experiments.

First, the *Pme*I-linearized plasmids pPICZA-Pdi and pPICZA (control) were separately introduced into GS115/pAOα-nROL (*n* = 1, 2, 3, …5). Twenty colonies of each strain (GS115/nROL-Pdi, *n* = 1, 2, 3, …5) were picked out for shake-flask fermentation. ROL activity was determined after 96 h methanol induction (Figure 1). To ensure that only one copy of pPICZA-Pdi was inserted into the genomic DNA of each transformants, the gene copy number of the strains was measured by RT-qPCR. As expected, co-expression of Pdi increased the level of ROL secretion. Interestingly, it seemed that as *ROL* gene copy number increased, the effect of Pdi on the secretion of ROL was less pronounced.

Next, to understand whether increasing the copy number of *Pdi* gene could further increase the expression level of ROL, the plasmid pPIC3.5K-Pdi was linearized by *Sac*I and introduced into GS115/nROL-Pdi (*n* = 1, 2, 3, …5) via electroporation. After determining the gene copy number via RT-qPCR, the strains GS115/nROL-2Pdi (*n* = 1, 2, 3, …5) harboring two copies of *Pdi* gene were cultured in shaking flasks. Surprisingly, when *Pdi* gene copy number further increased from one to two, ROL expression level no longer increased and, even, decreased (Figure 1). The gene copy number of the recombinant strains is presented in Table 1.

### 2.2. Effect of Co-Expressing a Single Helper Gene on ROL Expression

**Figure 2:**
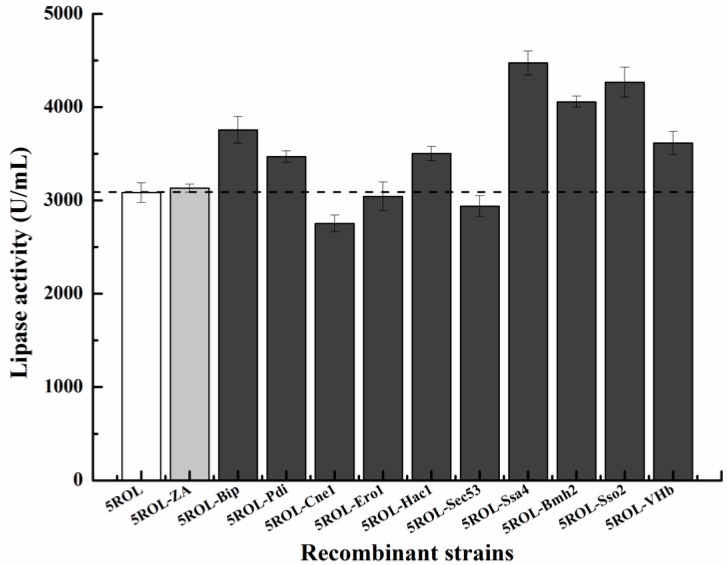
Effect of co-expressing helper proteins on ROL production in the strain GS115/pAOα-5ROL 11#. The dotted line indicates the lipase activity of the initial strain GS115/pAOα-5ROL 11#. All values are presented as the mean ± standard deviation of three independent experiments.

To investigate the influence of co-expressing a single helper gene on ROL expression, pPICZA (control) and pPICZA-derived plasmids harboring different helper genes were firstly electrotransformed into the strain GS115/pAOα-5ROL 11#. As shown in Figure 2, the co-expression of Ssa4, Bmh2, Sso2, Pdi, Bip, Hac1, and VHb showed positive effects on ROL production. Among them, the overexpression of Ssa4, Sso2, Bmh2, and Bip resulted in 45%, 39%, 32%, and 22% enhancement of ROL secretion level, respectively. The recombinant strain GS115/5ROL-Ssa4 12# displayed the maximum ROL activity at 4470 U/mL. However, several helper factors, such as Ero1, Sec53, and Cne1, did not improve ROL activity in the strain GS115/pAOα-5ROL 11#.

### 2.3. Co-Expressing Multiple Helper Genes to Enhance ROL Secretion

**Figure 3:**
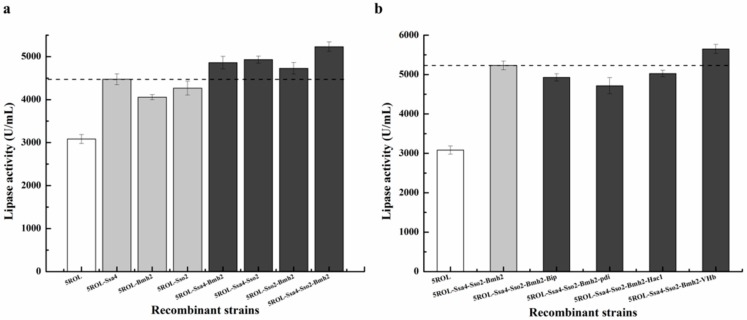
Cultivation properties of the recombinant strains in shaking flasks. (**a**) Effect of co-expressing multiple trafficking-related genes on ROL production in the strain GS115/pAOα-5ROL 11#. The dotted line indicates the lipase activity of the strain GS115/5ROL-Ssa4 12#. (**b**) Effect of co-expressing the helper proteins Bip, Pdi, Hac1, and VHb on ROL production in the strain GS115/5ROL-Ssa4-Sso2-Bmh2 4#. The dotted line indicates the lipase activity of the strain GS115/5ROL-Ssa4-Sso2-Bmh2 4#. Data represent the mean ± standard deviation of three independent experiments.

Based on the above results, co-expression of helper genes (Ssa4, Bmh2, and Sso2) participating in the protein secretion process significantly improved ROL production, suggesting that the efficiency of protein trafficking through the secretory pathway of the strain GS115/pAOα-5ROL 11# might greatly limit ROL production. To achieve even higher ROL production, the helper genes Ssa4, Bmh2, and Sso2 were first co-expressed together with ROL in GS115/pAOα-5ROL 11#. The plasmids pPIC3.5K-Ssa4-Bmh2, pPIC3.5K-Ssa4-Sso2, pPIC3.5K-Sso2-Bmh2, and pPIC3.5K-Ssa4-Sso2-Bmh2, which harbored multiple helper gene expression cassettes, were linearized by *Sal*I and then electroporated into the strain GS115/pAOα-5ROL 11#. The lipase activities of the obtained transformants were investigated in shaking flasks (Figure 3a). The activities of the recombinant strains GS115/5ROL-Sso2-Bmh2 13#, GS115/5ROL-Ssa4-Bmh2 18#, and GS115/5ROL-Ssa4-Sso2 11# respectively reached 4730 U/mL, 4860 U/mL, and 4930 U/mL, values that were all higher than those of the strains co-expressing only one helper gene. In addition, the highest ROL activity of 5230 U/mL was obtained in the strain GS115/5ROL-Ssa4-Sso2-Bmh2 4#, indicating that Ssa4, Bmh2, and Sso2 had a synergistic effect on ROL secretion.

In an attempt to further increase ROL expression level, other helper genes that also showed a positive effect on ROL expression, namely, *Pdi*, *Bip*, *Hac1*, and *VHb*, were also co-expressed with ROL in the strain GS115/5ROL-Ssa4-Sso2-Bmh2 4#. After induction for 96 h by methanol in shaking flasks, the fermentation supernatants of the recombinants were collected for the lipase assay. As shown in Figure 3b, only co-expression of VHb led to a slight improvement of ROL production. The lipase activity of the highest secreting strain, GS115/5ROL-Ssa4-Sso2-Bmh2-VHb 9#, reached 5650 U/mL, which was 83% higher than that obtained in GS115/pAOα-5ROL 11#.

### 2.4. High-Cell-Density Fermentation in a 3 L Bioreactor

**Figure 4:**
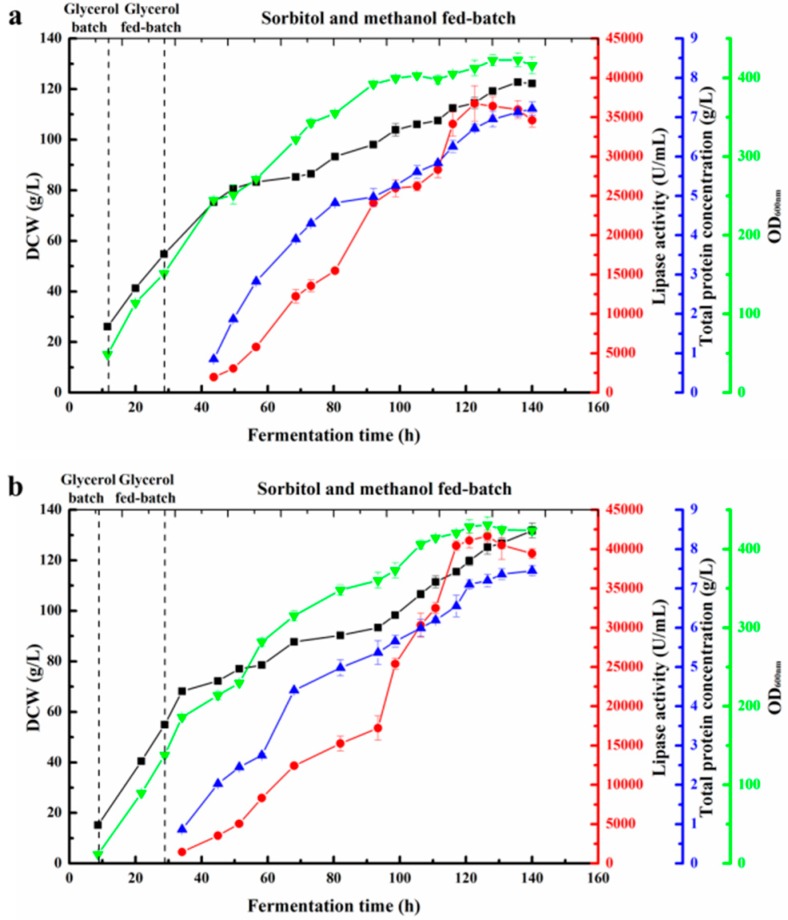
Time course of dry cell weight (DCW) (black square), ROL activity (red circle), total protein concentration (blue triangle), and optical density at 600 nm (OD_600nm_) (green inverted triangle) during a 3 L high-density fermentation of the strains (**a**) GS115/5ROL-Ssa4-Sso2-Bmh2 4# and (**b**) GS115/5ROL-Ssa4-Sso2-Bmh2-VHb 9#. Data represent the mean ± standard deviation of three independent experiments.

To obtain an even higher ROL production, the engineered strain GS115/5ROL-Ssa4-Sso2-Bmh2 4# was cultivated in a 3 L fermenter via a sorbitol–methanol co-feeding strategy [28]. The dissolved oxygen value was controlled at 20–50%. As shown in Figure 4a, the lipase activity of the strain GS115/5ROL-Ssa4-Sso2-Bmh2 4# achieved the maximum value after 122 h of fermentation, reaching 36,700 U/mL, which was 79% higher than that observed in the initial strain GS115/pAOα-5ROL 11# [14]. In addition, the ROL expression level of the strain GS115/5ROL-Ssa4-Sso2-Bmh2 4# was even higher than that of the strain co-expressing two ERAD-related genes in GS115/5ROL-Hrd1-Ubc1 1# (33,900 U/mL) [14]. Furthermore, the total protein concentration increased to 7.22 g/L, and the highest dry cell weight (DCW) and the optical density at 600 nm (OD_600nm_) reached 123 g/L and 422, respectively. These results indicate that the helper factors Ssa4, Bmh2, and Sso2 had a great positive effect on ROL expression in *P. pastoris*.

**Figure 5:**
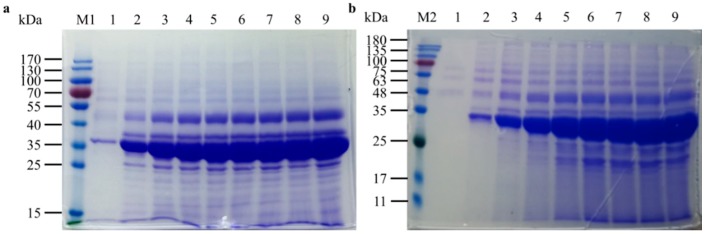
Time course of sodium dodecyl sulfate polyacrylamide gel electrophoresis (SDS-PAGE) analyses of extracellular proteins during high-density cultivation in a 3 L fermenter of the following strains: (**a**) GS115/5ROL-Ssa4-Sso2-Bmh2 4#: lanes 1–9, 12.5 µL of culture supernatant in the 3 L fermenter after 43, 50, 68, 73, 92, 99, 105, 116, and 128 h of fermentation, respectively. Lane M1, low-molecular-weight marker; (**b**) GS115/5ROL-Ssa4-Sso2-Bmh2-VHb 9#: lanes 1–9, 12.5 µL of culture supernatant in the 3 L fermenter after 29, 45, 68, 82, 93, 110, 117, 126, and 140 h of cultivation, respectively. Lane M2, low-molecular-weight marker.

To investigate the effect of VHb on ROL production, a fed-batch study of GS115/5ROL-Ssa4-Sso2-Bmh2-VHb 9# was conducted under the same cultivation conditions. The results showed that co-expression of VHb further improved ROL activity to 41,700 U/mL and increased the total protein concentration to 7.45 g/L after 126 h of cultivation (Figure 4b). The lipase activity was 14% higher than that of GS115/5ROL-Ssa4-Sso2-Bmh2 4#. In addition, compared with the strain GS115/5ROL-Ssa4-Sso2-Bmh2 4#, further co-expression of VHb with ROL slightly increased cell growth, and DCW and OD_600nm_ increased to 132 g/L and 431, respectively, at the end of the fermentation. Sodium dodecyl sulfate polyacrylamide gel electrophoresis (SDS-PAGE) analyses of the supernatants during high-density cultivation are shown in Figure 5.

## 3. Discussion

ROL, an important industrial lipase, is often used to produce biodiesel [6]. In order to reduce the cost of industrial applications, several researchers have employed multiple molecular genetic strategies and fermentation techniques to increase its production level [7,8,14]. Yu et al. [29] employed an approach consisting in replacing the pro-peptide of ROL with *Rhizopus chinensis* lipase pro-peptide to promote ROL expression, and the maximal lipase activity reached 4050 U/mL in a 7 L fermenter. Besides, ROL production yield was further enhanced via co-expression of Ero1 and Pdi, followed by optimization of the NH_4_^+^ concentration in a high-density fermentation environment, and ROL activity attained 12,019 U/mL [8]. In addition, intracellular co-expression of the *S. cerevisiae* UPR transcription factor Hac1 as well as damage of the *Gas1* gene resulted in about a seven-fold enhancement in the production level of ROL [7]. Moreover, ROL expression was improved 15.8-fold by combining the optimization of gene copy number with the co-expression of ERAD-related proteins, reporting the highest level of 33,900 U/mL in a 3 L fermenter [14]. However, the production level of ROL should be further increased to meet the huge industrial demands. In the present study, more strategies have been attempted and successfully provided a higher ROL activity.

Many researchers have reported that the overexpression of exogenous genes in *P. pastoris* might result in the accumulation of unfolded proteins that cause ER stress and activate the UPR [16,17,30]. Therefore, overloading foreign proteins might represent an important bottleneck for high-level protein expression. To alleviate this problem and obtain even higher protein productions, several strategies focused on aiding protein folding by co-producing the UPR activator Hac1 or co-overexpressing ER chaperones, such as Ero1, Pdi, and Bip [7,13,20,21]. Additionally, the production level of foreign genes could also be improved by co-overexpression of proteins involved in transport pathways [20]. However, the same strategy might have different effects on various target proteins and in various strains. In addition, it is not clear which chaperone or helper protein may be helpful in improving ROL expression. Moreover, although the copy number of exogenous genes is one of the key parameters to consider when trying to optimize protein production in *P. pastoris*, it is not certain that the copy number of helper genes is equally important. Therefore, before attempting to co-express multiple potential helper proteins with ROL to increase its expression level, the important ER chaperone Pdi was selected as a representative chaperone to investigate the effect of different copies of a helper gene on ROL production level.

Firstly, a series of strains harboring different copies of *ROL* gene and different copies of *Pdi* gene were generated via two rounds of electrotransformation. As shown in Figure 1, the expression levels of ROL were obviously improved by co-expression of Pdi. However, it can also be seen that ROL activity increased slightly with the increment of *ROL* gene copies. Moreover, compared with the strains co-expressing one copy of *Pdi* gene, increasing the dosage of *Pdi* gene in recombinant strains resulted in a very little effect on ROL secretion, even in a negative effect. These results are similar to those previously reported [13]. A possible explanation is that the gene transcription level under the control of pAOX1 was already very high, and a single copy of *Pdi* gene was sufficient to aid protein folding. Besides, the strains harboring multiple copies of *Pdi* gene would produce higher levels of Pdi proteins, which would compete with the target proteins for transcription, translation, and other secretion processes. Additionally, it was reported by Schwarzhans et al. [31] that a higher gene copy number does not necessarily result in high production.

On the basis of the above results, the strain with multiple copies of a helper gene is not necessary for the purpose of enhancing ROL production level. Moreover, multiple plasmid integration events occur naturally at an extremely low frequency (~1%) during the transformation process in *P. pastoris* [32]. Hence, when investigating the effect of co-expressing helper proteins on ROL activity, 20 colonies of each strain were randomly selected, without a post-transformational vector amplification (PTVA) [33,34] process, to achieve this goal, and these colonies would obviously include the single-copy transformants. As shown in Figure 2, individual co-overexpression of Ssa4, Sso2, Bmh2, and Bip with ROL greatly increased ROL expression, to levels about 45%, 39%, 32%, and 22% higher, respectively, than that of the initial strain GS115/pAOα-5ROL 11#. Among them, co-expression of Sso2 with ROL in strain GS115/5ROL-Ssa4 12# produced the maximum ROL activity of 4470 U/mL. Besides, co-expressing Pdi, Hac1, and VHb individually also had positive effects on ROL production. However, Ero1, Sec53, and Cne1 did not improve the ROL expression level. These results are in contrast with those reported, which showed that the overexpression of Ero1 increased the production of *Candida rugosa* lipase Lip1 in *P. pastoris* [21] and the co-production of Sec53 and Cne1 greatly enhanced the expression level of GOD [20], suggesting that the role of helper genes strongly depended on the target protein or the target strain. Hence, it is worth screening different key helper genes for the purpose of increasing a target protein′s secretion level.

The results of co-expressing a single helper protein with ROL indicated that the genes *Ssa4*, *Bmh2*, and *Sso2*, which are involved in vesicular trafficking, had a significant influence on ROL production. To explore whether co-expression of multiple trafficking-related genes could further enhance the ROL expression level, pPIC3.5K-derived plasmids harboring multiple trafficking-related genes were constructed and electrotransferred into the strain GS115/pAOα-5ROL 11# to achieve this goal. As shown in Figure 3a, the trafficking-related genes did have a synergistic effect on ROL production as expected, with ROL activities reaching 4730 U/mL, 4860 U/mL, and 4930 U/mL in the recombinant strains GS115/5ROL-Sso2-Bmh2 13#, GS115/5ROL-Ssa4-Bmh2 18#, and GS115/5ROL-Ssa4-Sso2 11#, respectively. Moreover, co-expression of Sso2, Bmh2, and Ssa4 with ROL in the strain GS115/5ROL-Ssa4-Sso2-Bmh2 4# produced the highest ROL activity of 5230 U/mL in shake-flask fermentation, which was about 70% higher than that obtained in the initial strain GS115/pAOα-5ROL 11#. These results imply that protein trafficking through the transport pathway might be a bottleneck for high-level expression of ROL in the strain of GS115/pAOα-5ROL 11#.

To further increase the ROL production level, the remnant helper proteins (Bip, Pdi, Hac1, and VHb), which also showed a positive effect on ROL expression, were respectively co-expressed with ROL in strain GS115/5ROL-Ssa4-Sso2-Bmh2 4# via a new round of electroporation. Unfortunately, the results of shake-flask fermentation demonstrated that co-expression of these helper factors (Bip, Pdi, and Hac1, which are involved in improving protein folding efficiency) did not further improve ROL expression level (Figure 3b). These results might be due to the fact that ROL production in strain GS115/5ROL-Ssa4-Sso2-Bmh2 4# is improved via enhancing protein trafficking, and, as the efficiency of the protein transport process increases, the proteins would leave the ER faster, which might then help cells reduce ER stress; subsequently, the expression of ROL might present a new bottleneck, so that a continuous increase of the protein folding function to reduce ER stress becomes less effective. However, co-expression of VHb along with ROL in the recombinant strain GS115/5ROL-Ssa4-Sso2-Bmh2-VHb 9# slightly improved ROL activity to 5650 U/mL, which is about an 83% improvement compared to the initial strain (GS115/pAOα-5ROL 11#) and might be attributed to the fact that VHb could promote cell growth in an environment with a high cell density.

High-density fermentation of strains GS115/5ROL-Ssa4-Sso2-Bmh2 4# and GS115/5ROL-Ssa4-Sso2-Bmh2-VHb 9# were performed in a 3 L fermenter via a sorbitol–methanol co-feeding strategy (Figure 4). Co-expression of Ssa4, Bmh2, and Sso2 with ROL in strain GS115/5ROL-Ssa4-Sso2-Bmh2 4# significantly enhanced ROL production level, the lipase activity reached 36,700 U/mL, and the total protein concentration attained 7.22 g/L. Moreover, when VHb was co-expressed with ROL in strain GS115/5ROL-Ssa4-Sso2-Bmh2-VHb 9#, ROL activity and protein concentration further increased to 41,700 U/mL and 7.45 g/L. Additionally, the maximum DCW (132 g/L) and OD_600nm_ (431) were also slightly higher than those in the strain GS115/5ROL-Ssa4-Sso2-Bmh2 4#. These results indicated that co-expression of VHb could exert a positive effect on cell growth and, in return, improve the secretion level of ROL. Overall, the combined strategies used in this study resulted in a twofold increase in ROL expression compared to the initial strain GS115/pAOα-5ROL 11# (20,500 U/mL) [14].

## 4. Materials and Methods

### 4.1. Strains, Plasmids, and Media

*P. pastoris* GS115, *Escherichia coli* Top10 cells, and the plasmids pPIC3.5K and pPICZA were bought from Invitrogen (Carlsbad, CA, USA). Recombinant *P. pastoris* strains GS115/pAOα-nROL (*n* = 1, 2, 3, …5) were constructed previously [14]. The plasmid pPIC3.5K-VHb was stored in our laboratory [26]. The pMD19-T simple vector, PrimeStar HS DNA polymerase, restriction enzymes, and T4 DNA ligase were purchased from TaKaRa (Dalian, China). Real-time quantitative PCR (RT-qPCR) reagents and materials were commercially obtained from Tiangen Biotechnology (Beijing, China). All strains, plasmids, and primers used in this study are listed in Table 2 and Table 3.

The media Luria–Bertani (LB), low-salt LB, yeast extract-peptone-dextrose (YPD), buffered methanol-complex medium (BMMY), buffered glycerol-complex medium (BMGY), BMMY-rhodamine B-olive oil medium (BRBO), and the culture conditions were as described previously [14].

### 4.2. Vector Construction

The helper genes *Ssa4* and *Bip* were amplified from the genomic DNA of *P. pastoris* strain GS115; the polymerase chain reaction (PCR) fragments were double-digested with *Xho*I and *Not*I, then inserted into *Xho*I/*Not*I-digested pPICZA to generate pPICZ-Ssa4 and pPICZ-Bip, respectively.

The genes *Cne1*, *Ero1*, and *Sec53* were respectively cloned using the primer pairs Cne1-F/Cne1-R, Ero1-F/Ero1-R, and Sec53-F/Sec53-R, which all included *Asu*II/*Not*I-restriction sites at both ends and the chromosomal DNA of GS115 as the template. The PCR segments were digested with *Asu*II and *Not*I then transferred into pPICZA, respectively forming pPICZ-Cne1, pPICZ-Ero1, and pPICZ-Sec53.

To clone the *Bmh2* gene and synchronously eliminate the *EcoR*I restriction site via synonymous mutation, the upstream and downstream parts of the *Bmh2* gene were respectively cloned from the genomic DNA of GS115 using the primer pairs Bmh2-F/bm-r and bm-f/Bmh2-R. These two PCR products were purified, then mixed as overlap extension PCR (OE-PCR) templates, and the OE-PCR was conducted using the primer pair Bmh2-F/Bmh2-R, generating the *Bmh2* gene. In order to obtain the *Sso2* gene and synchronously remove the *Sal*I site in the *Sso2* gene, and to clone the *Hac1* gene without the intron part, the *Sso2* and *Hac1* genes were amplified by OE-PCR as described for *Bmh2*. Additionally, the *VHb* gene and *Pdi* gene were respectively amplified by PCR using the primers VHb-F/VHb-R and Pdi-F/Pdi-R. These five gene fragments were double-digested with *EcoR*I and *Not*I and then cloned into the *EcoR*I/*Not*I-opened plasmid pPICZA, respectively generating pPICZ-Bmh2, pPICZ-Sso2, pPICZ-Hac1, pPICZ-VHb, and pPICZ-Pdi.

To generate the pPIC3.5K-derived vectors, the helper gene fragments were obtained by double-digesting the pPICZA-derived plasmids with *Sac*I and *Not*I and then inserting them into the *Sac*I/*Not*I-treated vector pPIC3.5K. Additionally, the plasmids harboring multiple expression-related helper gene cassettes were constructed on the basis of the pPIC3.5K-derived expression vector, according to a previously described in vitro multi-copy vector construction method [14]. All plasmids were verified by DNA sequencing at Tsingke Biological Technology Co. (Wuhan, China).

### 4.3. Transformation of P. Pastoris

The plasmids were linearized using appropriate restriction enzymes before transformation. To prepare *P. pastoris* competent cells, an overnight culture of the host strain was grown in a 250 mL flask containing 100 mL YPD medium at 28 °C until the cell optical density at 600nm (OD_600nm_) reached 1.2–1.5. The cells were then harvested by centrifugation at 2000× *g* for 4 min and washed twice in 100 mL of pre-cooled sterile water. Subsequently, the pellet was resuspended twice in 20 mL of sterilized and cold-processed 1 M sorbitol solution, followed by resuspension in 200 µL of 1 M sorbitol. About 2 µg of the linearized plasmids were added to 80 µL competent cells, then transferred into a new pre-cooled 2 mm electroporation cuvette (Invitrogen) for 10 min on ice. The cuvette was pulsed for about 5 ms using a Gene Pulser apparatus (Bio-Rad, Hercules, CA, USA) with the operating parameters of 1.5 kV, 200 Ω, and 25 µF. Then, 1 mL of 1 M sorbitol was added immediately and the cells were incubated for 1–2 h at 30 °C. The transformants were selected on YPD plates containing 100 µg/mL zeocin or 200 µg/mL geneticin (G418) for 2–3 days at 28 °C.

### 4.4. Recombinant Screening and Shake-Flask Culture

Transformants with zeocin or G418 resistance were randomly picked onto BRBO plates. Soon afterwards, 200 µL of absolute methanol was added every day to the culture dish. Twenty colonies with clear transparent circles were selected for incubation into a 500 mL flask containing 20 mL of BMGY medium. About one day later, the cells were gathered by centrifugation and transferred into 20 mL of BMMY at 27 °C. Additionally, 1.2% (*v*/*v*) methanol was added daily to the flask to induce ROL expression.

### 4.5. Enzyme Assay and Total Protein Concentration

ROL activity was determined via a titrimetry method. Four mL of substrate (250 mL/L olive oil emulsified with 20 g/L polyvinyl alcohol solution), 5 mL of 50 mM Tris-HCl buffer (pH 8.0), and 1 mL of properly diluted fermentation filtrate were mixed in a 50 mL flask. After incubation in a shaking water bath at 35 °C for 10 min, 15 mL of absolute ethanol/acetone (1:1, *v*/*v*) was added to the mixture to stop the reaction process. The amount of released fatty acids was determined by titration with 50 mM NaOH. One unit (U) of ROL activity was defined as the amount of lipase producing 1 µmol of fatty acid from the substrate per 1 min under the hydrolysis conditions. Total protein concentration was measured as described by Bradford, using bovine serum albumin as a standard [35]. Each experiment was repeated three times.

### 4.6. Gene-Copy Number Determination by RT-qPCR

Absolute quantification to determine the gene copy number was carried out by RT-qPCR, as described previously [14]. RT-qPCR reactions were performed using a StepOnePlus instrument with StepOne software version 2.3 (Applied Biosystems, Foster City, CA, USA). The gene copy number was calculated via absolute quantification by the method described by Abad et al. [36].

### 4.7. 3 L Fermenter Cultivation

The high-density fermentation was executed in a 3 L fermenter (BIOTECH-3BG-7000A; Baoxing Co., Shanghai, China). The fermentation process control and cultivation conditions followed a procedure established previously [14].

### 4.8. Dry Cell Weight (DCW) and OD_600nm_

The DCW (g/L) was determined by centrifuging 10 mL of fermentation broth in a pre-weighted tube at 5000× *g* for 10 min, followed by drying the samples at 105 °C to a constant. The samples were diluted with sterile water to measure the OD_600nm_.

### 4.9. Sodium Dodecyl Sulfate Polyacrylamide Gel Electrophoresis (SDS-PAGE) Analysis

The polyacrylamide gel consisted of two parts: a 6% stacking gel and a 12% separating gel. SDS-PAGE analysis was conducted on a vertical mini gel apparatus (Bio-Rad, Hercules, CA, USA). The samples were stained with Coomassie Brilliant Blue R-250 (Amresco, Solon, OH, USA).

## 5. Conclusions

A series of recombinant strains containing different gene copy numbers of *ROL* gene and *Pdi* gene were first constructed to investigate the effects of *Pdi* gene dosage on ROL production. The results showed that one copy of *Pdi* gene was enough for a high-level expression of ROL. On the basis of this observation, to study the effect of the co-expression of helper genes on ROL expression, we only generated strains harboring one copy of the helper genes by screening 20 recombinant colonies directly after electroporation, without a PTVA process. Some helper proteins were identified to have positive effects on ROL expression. The helper proteins Ssa4, Bmh2, and Sso2, all participating in vesicular trafficking, could strongly promote ROL expression; when combined, a further improvement in ROL production level to 5230 U/mL was obtained in the strain GS115/5ROL-Ssa4-Sso2-Bmh2 4#. Among the remaining helper genes, which also showed a positive effect on ROL production, only the co-expression of VHb could further slightly increase ROL secretion to 5650 U/mL in the strain of GS115/5ROL-Ssa4-Sso2-Bmh2-VHb 9#. Furthermore, fed-batch studies of the two optimal strains were conducted in a 3 L fermenter, and the maximum ROL activity obtained was 41,700 U/mL, the highest level ever reported. This study suggests that screening potential helper factors and then combining them together in a reasonable way is a feasible approach to increase a target protein expression to an extremely high level in *P. pastoris*.

## Figures and Tables

**Table 1:** Recombinant strains with different copy numbers of the *Pdi* gene and *ROL* gene ^1^.

Strains	Gene Copy Number	
	*ROL*	*Pdi*
GS115 (control)	0	1
GS115/ROL-Pdi	1	2
GS115/2ROL-Pdi	2	2
GS115/3ROL-Pdi	3	2
GS115/4ROL-Pdi	4	2
GS115/5ROL-Pdi	5	2
GS115/ROL-2Pdi	1	3
GS115/2ROL-2Pdi	2	3
GS115/3ROL-2Pdi	3	3
GS115/4ROL-2Pdi	4	3
GS115/5ROL-2Pdi	5	3

^1^ The copy number of *Pdi* minus one (endogenous gene) is the copy number of the inserted gene.

**Table 2:** Strains and plasmids used in this study.

Plasmids/Strains	Description	References
pPICZA	Intracellular expression vector, *Sh ble* gene (zeocin ^r^)	Invitrogen
pPIC3.5K	Intracellular expression vector, *His4* gene and *Kan* gene (G418 ^r^)	Invitrogen
pPIC3.5K-VHb	pPIC3.5K derivative, carrying *VHb* gene expression cassette	[26]
pPICZ-Ssa4	pPICZA derivative, carrying *Ssa4* gene expression cassette	This study
pPICZ-Bip	pPICZA derivative, carrying *Bip* gene expression cassette	This study
pPICZ-Cne1	pPICZA derivative, carrying *Cne1* gene expression cassette	This study
pPICZ-Ero1	pPICZA derivative, carrying *Ero1* gene expression cassette	This study
pPICZ-Sec53	pPICZA derivative, carrying *Sec53* gene expression cassette	This study
pPICZ-Bmh2	pPICZA derivative, carrying *Bmh2* gene expression cassette	This study
pPICZ-Sso2	pPICZA derivative, carrying *Sso2* gene expression cassette	This study
pPICZ-Hac1	pPICZA derivative, carrying *Hac1* gene expression cassette	This study
pPICZ-Pdi	pPICZA derivative, carrying *Pdi* gene expression cassette	This study
pPICZ-VHb	pPICZA derivative, carrying *VHb* gene expression cassette	This study
pPIC3.5K-Pdi	pPIC3.5K derivative, carrying *Pdi* gene expression cassette	This study
pPIC3.5K-Ssa4-Sso2	pPIC3.5K derivative, carrying *Ssa4* and *Sso2* expression cassettes	This study
pPIC3.5K-Ssa4-Bmh2	pPIC3.5K derivative, carrying *Ssa4* and *Bmh2* expression cassettes	This study
pPIC3.5K-Sso2-Bmh2	pPIC3.5K derivative, carrying *Sso2* and *Bmh2* expression cassettes	This study
pPIC3.5K-Ssa4-Sso2-Bmh2	pPIC3.5K derivative, carrying *Ssa4*, *Sso2*, and *Bmh2* expression cassettes	This study
*E. coli* Top10	*F-*, *mcrAΔ*(*mrr-hsd RMS-mcrBC*), ϕ80, *lacZΔM15*, *ΔlacX74*, *recA1*, *araΔ139Δ(ara-leu)7697*, *galU*, *galK*, *rps*, (*StrR*) *endA1*, *nupG*	Invitrogen
*P. pastoris* GS115	Host strain (his4^−^, Mut^+^)	Invitrogen
GS115/pAOα-ROL	GS115 harboring one copy of *ROL* gene	[14]
GS115/pAOα-2ROL	GS115 harboring two copies of *ROL* gene	[14]
GS115/pAOα-3ROL	GS115 harboring three copies of *ROL* gene	[14]
GS115/pAOα-4ROL	GS115 harboring four copies of *ROL* gene	[14]
GS115/pAOα-5ROL	GS115 harboring five copies of *ROL* gene	[14]
GS115/ROL-Pdi	GS115/pAOα-ROL harboring pPICZ-Pdi	This study
GS115/2ROL-Pdi	GS115/pAOα-2ROL harboring pPICZ-Pdi	This study
GS115/3ROL-Pdi	GS115/pAOα-3ROL harboring pPICZ-Pdi	This study
GS115/4ROL-Pdi	GS115/pAOα-4ROL harboring pPICZ-Pdi	This study
GS115/5ROL-Pdi	GS115/pAOα-5ROL harboring pPICZ-Pdi	This study
GS115/ROL-2Pdi	GS115/pAOα-ROL harboring pPICZ-Pdi and pPIC3.5K-Pdi	This study
GS115/2ROL-2Pdi	GS115/pAOα-2ROL harboring pPICZ-Pdi and pPIC3.5K-Pdi	This study
GS115/3ROL-2Pdi	GS115/pAOα-3ROL harboring pPICZ-Pdi and pPIC3.5K-Pdi	This study
GS115/4ROL-2Pdi	GS115/pAOα-4ROL harboring pPICZ-Pdi and pPIC3.5K-Pdi	This study
GS115/5ROL-2Pdi	GS115/pAOα-5ROL harboring pPICZ-Pdi and pPIC3.5K-Pdi	This study
GS115/5ROL-Ssa4	GS115/pAOα-5ROL harboring pPICZ-Ssa4	This study
GS115/5ROL-Bip	GS115/pAOα-5ROL harboring pPICZ-Bip	This study
GS115/5ROL-Cne1	GS115/pAOα-5ROL harboring pPICZ-Cne1	This study
GS115/5ROL-Ero1	GS115/pAOα-5ROL harboring pPICZ-Ero1	This study
GS115/5ROL-Sec53	GS115/pAOα-5ROL harboring pPICZ-Sec53	This study
GS115/5ROL-Bmh2	GS115/pAOα-5ROL harboring pPICZ-Bmh2	This study
GS115/5ROL-Sso2	GS115/pAOα-5ROL harboring pPICZ-Sso2	This study
GS115/5ROL-Hac1	GS115/pAOα-5ROL harboring pPICZ-Hac1	This study
GS115/5ROL-VHb	GS115/pAOα-5ROL harboring pPICZ-VHb	This study
GS115/5ROL-Sso2-Bmh2	GS115/pAOα-5ROL harboring pPIC3.5K-Sso2-Bmh2	This study
GS115/5ROL-Ssa4-Bmh2	GS115/pAOα-5ROL harboring pPIC3.5K-Ssa4-Bmh2	This study
GS115/5ROL-Ssa4-Sso2	GS115/pAOα-5ROL harboring pPIC3.5K-Ssa4-Sso2	This study
GS115/5ROL-Ssa4-Sso2-Bmh2	GS115/pAOα-5ROL harboring pPIC3.5K-Ssa4-Sso2-Bmh2	This study
GS115/5ROL-Ssa4-Sso2-Bmh2-Bip	GS115/pAOα-5ROL harboring pPIC3.5K-Ssa4-Sso2-Bmh2 and pPICZ-Bip	This study
GS115/5ROL-Ssa4-Sso2-Bmh2-Pdi	GS115/pAOα-5ROL harboring pPIC3.5K-Ssa4-Sso2-Bmh2 and pPICZ-Pdi	This study
GS115/5ROL-Ssa4-Sso2-Bmh2-Hac1	GS115/pAOα-5ROL harboring pPIC3.5K-Ssa4-Sso2-Bmh2 and pPICZ-Hac1	This study
GS115/5ROL-Ssa4-Sso2-Bmh2-VHb	GS115/pAOα-5ROL harboring pPIC3.5K-Ssa4-Sso2-Bmh2 and pPICZ-VHb	This study

**Table 3:** Primers used for PCR in this study.

Primers	Sequence (5′–3′)	Annotation	GenBank
Ssa4-F	CCGCTCGAGACGATGGGTAAATCAATTGGAATTG	*Xho*I site (underlined)	XM_002492398.1
Ssa4-R	ATTTGCGGCCGCTTAATCGACTTCTTCCACGG	*Not*I site (underlined)	
Bip-F	CCGCTCGAGACGATGCTGTCGTTAAAACCATCTTGG	*Xho*I site (underlined)	AY965684.1
Bip-R	ATTTGCGGCCGCCTACAACTCATCATGATCATAGTCA	*Not*I site (underlined)	
Cne1-F	ATTATTCGAAACGATGAAGATCTCTACCATTGC	*Asu*II site (underlined)	XM_002491173.1
Cne1-R	ATTTGCGGCCGCCTAGGTTCTCTTTGTAGC	*Not*I site (underlined)	
Ero1-F	ATTATTCGAAACGATGAGGATAGTAAGGAGCG	*Asu*II site (underlined)	XM_002489600.1
Ero1-R	ATTTGCGGCCGCTTACAAGTCTACTCTATATG	*Not*I site (underlined)	
Sec53-F	ATTATTCGAAACGATGTCGTTTTCTAATAAAGAAGATCC	*Asu*II site (underlined)	XM_002492115.1
Sec53-R	ATTTGCGGCCGCTTACAGGGAAAAGAGCTCC	*Not*I site (underlined)	
Bmh2-F	CTGAATTCACGATGTCAAGAGAAGATTCTG	*EcoR*I site (underlined)	XM_002490942.1
Bmh2-R	ATTTGCGGCCGCTCACTCTTCATCTTTGGGAG	*Not*I site (underlined)	
bm-f	GTTATTTGGCCGAATTTGCTG	Elimination of *EcoR*I site	
bm-r	CAGCAAATTCGGCCAAATAAC		
Sso2-F	CTGAATTCACGATGAGTAACCAGTATAATCC	*EcoR*I site (underlined)	XM_002490368.1
Sso2-R	ATTTGCGGCCGCCTATCTTCCCCAGTTTCCG	*Not*I site (underlined)	
sso-f	CTGAGACCAGTCGTCAACG	Elimination of *Sal*I site	
sso-r	CGTTGACGACTGGTCTCAG		
Hac1-F	CTGAATTCATGCCCGTAGATTCTTCTC	*EcoR*I site (underlined)	XM_002489994.1
Hac1-R	ATTTGCGGCCGCCTATTCCTGGAAGAATACAAAGTC	*Not*I site (underlined)	
ha-f	AATCGGTTGCATCATCCAGCAGCACCATTTACCGCTAATGCA		
ha-r	TGCATTAGCGGTAAATGGTGCTGCTGGATGATGCAACCGATT		
VHb-F	CTGAATTCACCATGTTAGACCAGCAAACC	*EcoR*I site (underlined)	L21670.1
VHb-R	ATTTGCGGCCGCTTATTCAACCGCTTGAGCG	*Not*I site (underlined)	
Pdi-F	CTGAATTCATGCAATTCAACTGGAATATTAAAACTGTG	*EcoR*I site (underlined)	EU805807.1
Pdi-R	ATTTGCGGCCGCTTAAAGCTCGTCGTGAGCGTC	*Not*I site (underlined)	
qROL-F	CAAGTATGCTGGTATCGCTG	RT-PCR for *ROL*	
qROL-R	GAGTTGGTACCACGGAAAAC	RT-PCR for *ROL*	
qGADPH-F	CGGTGTTTTCACCACTTTGGA	RT-PCR for *GADPH* ^1^	XM_002491300.1
qGADPH-R	CAACGAACATTGGAGCATCCT	RT-PCR for *GADPH*	
qPdi-F	GCCGTTAAATTCGGTAAGCA	RT-PCR for *Pdi*	
qPdi-F	TCAGCTCGGTCACATCTTTG	RT-PCR for *Pdi*	

^1^ Glyceraldehyde-3-phosphate dehydrogenase gene of *P. pastoris*.

## Abbreviations

Bip	immunoglobulin-binding protein
BMGY	buffered glycerol-complex medium
BMMY	buffered methanol-complex medium
BRBO	BMMY-rhodamine B-olive oil medium
Cne1	calnexin-like protein
DCW	dry cell weight
ER	endoplasmic reticulum
ERAD	ER-associated degradation
Ero1	ER oxidoreduction 1
GOD	glucose oxidase
LB	Luria–Bertani
OD_600nm_	optical density at 600 nm
OE-PCR	overlap extension PCR
pAOX1	alcohol oxidase 1 promoter
Pdi	protein disulfide isomerase
PTVA	posttransformational vector amplification
ROL	*Rhizopus oryzae* lipase
SDS-PAGE	sodium dodecyl sulfate polyacrylamide gel electrophoresis
UPR	unfolded protein response
VHb	vitreoscilla hemoglobin
YPD	yeast extract-peptone-dextrose
